# Ultra-low-power hybrid light–matter solitons

**DOI:** 10.1038/ncomms9317

**Published:** 2015-09-24

**Authors:** P. M. Walker, L. Tinkler, D. V. Skryabin, A. Yulin, B. Royall, I. Farrer, D. A. Ritchie, M. S. Skolnick, D. N. Krizhanovskii

**Affiliations:** 1 Department of Physics and Astronomy, University of Sheffield, Hounsfield Road, S3 7RH Sheffield, UK; 2 Department of Physics, University of Bath, BA2 7AY Bath, UK; 3 ITMO University 197101, Kronverksky pr. 49, St. Petersburg, Russian Federation; 4 Cavendish Laboratory, University of Cambridge, J J Thomson Avenue, CB3 0HE Cambridge, UK

## Abstract

New functionalities in nonlinear optics will require systems with giant optical nonlinearity as well as compatibility with photonic circuit fabrication techniques. Here we introduce a platform based on strong light–matter coupling between waveguide photons and quantum-well excitons. On a sub-millimetre length scale we generate picosecond bright temporal solitons at a pulse energy of only 0.5 pJ. From this we deduce a nonlinear refractive index three orders of magnitude larger than in any other ultrafast system. We study both temporal and spatio-temporal nonlinear effects and observe dark–bright spatio-temporal polariton solitons. Theoretical modelling of soliton formation in the strongly coupled system confirms the experimental observations. These results show the promise of our system as a high speed, low power, integrated platform for physics and devices based on strong interactions between photons.

## Abstract (web summary)

Harnessing nonlinear optics in optoelectronic devices requires a platform that exhibits both giant optical nonlinearity and is compatible with photonic-circuit fabrication. Here, the authors demonstrate such a system that uses strong light–matter coupling between waveguide photons and quantum-well excitons.

## Graphical abstract

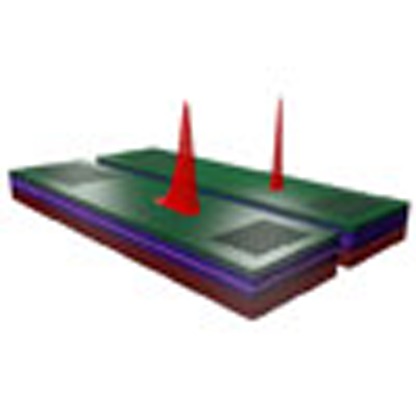

The introduction of strong nonlinearity into photonic circuits has many applications ranging from optical information processing^1^,^2^,^3^ and soliton physics^4^,^5^ to the study of inter-particle interactions^5^,^6^,^7^,^8^,^9^,^10^,^11^ in photonic analogues of important physical systems such as photonic topological insulators^12^,^13^,^14^,^15^, optical analogues of quantum hall systems.

Temporal optical solitons are one of the most fundamental nonlinear phenomena in optics. They arise owing to a competition between group velocity dispersion (GVD), where different frequency components accumulate different phase during propagation, and nonlinear phase modulation where intensity-dependent phase shifts can exactly balance those due to GVD^4^. They have been proposed for applications^4^ and observed^16^ in long-haul communications lines, and more recently investigated in the micro- and nanoscale waveguides suitable for design of all optical processing chips^17^,^18^,^19^,^20^,^21^. On-chip applications require that solitons form and interact over small distances, which requires that both the GVD and nonlinear effects develop on very short length scales. It is also important for information processing applications that the underlying nonlinearity responds on picosecond or faster timescales. Achieving short nonlinear length scales while minimizing power requirments necessitates very large nonlinearity. To generate significant GVD, photonic crystal band engineering and semiconductor photonic wires have been used in a number of schemes^17^,^18^,^19^,^20^,^21^.

Giant nonlinearity and GVD can be achieved simultaneously by exploiting the unique optical properties of exciton-polaritons^22^. These quasi-particles are the quantum eigenmodes formed when photons couple strongly to a quantum-well exciton resonance. They have characteristics coming from both their light and matter constituents, with the proportion depending on the detuning from resonance. The photonic component contributes long lifetime and detailed control over system properties via photonic circuit fabrication techniques^23^,^24^. Matter-like interparticle scattering leads to enormous nonlinearity, which responds on an ultrafast timescale.

In this work we address the problem of introducing strong optical nonlinearity by studying ultra-low-power optical solitons in a novel slab waveguide geometry we recently introduced^25^, where photons are strongly coupled to quantum-well excitons. We demonstrate formation of bright picosecond temporal polariton solitons with pulse energies of <0.5 pJ. From the soliton formation threshold, we deduce a nonlinear refractive index three orders of magnitude larger than in any other ultrafast optical system, which verifies the highly nonlinear nature of our platform. We show that the frequency-dependent transition of polaritons from photon-like to matter-like leads to giant GVD on the order of 1,000 ps^2^ m^−1^. This is comparable to that achieved in fibre Bragg gratings and photonic crystal waveguides^19^,^20^,^21^. As we achieve the GVD necessary for soliton formation without the need for transverse photonic confinement, we are able to study spatio-temporal effects as well as purely temporal ones. We experimentally demonstrate dark–bright spatio-temporal polariton solitons, which to our knowledge have not previously been seen in any experimental setting with either photons or polaritons. It is noteworthy that bright^26^,^27^ and spatial dark^28^ exciton-polariton solitons have previously been reported in strongly coupled Bragg microcavities. Compared with these, our system has the advantages of an order of magnitude faster soliton velocity, simplicity of design and fabrication, compatibility with existing designs of high-performance waveguide photonic circuit elements and, crucially, does not depend on external continuous wave pumping to balance loss for soliton formation to be possible. Apart from these, the major advantages of our platform for nonlinear optics are the combination of very large nonlinearity and ultra-fast temporal response. The intrinsic nature of the large dispersion is also advantageous for on-chip soliton formation, pulse compression^21^ and the study of two-dimensional effects.

## Results

### Experiment

**Figure 1:**
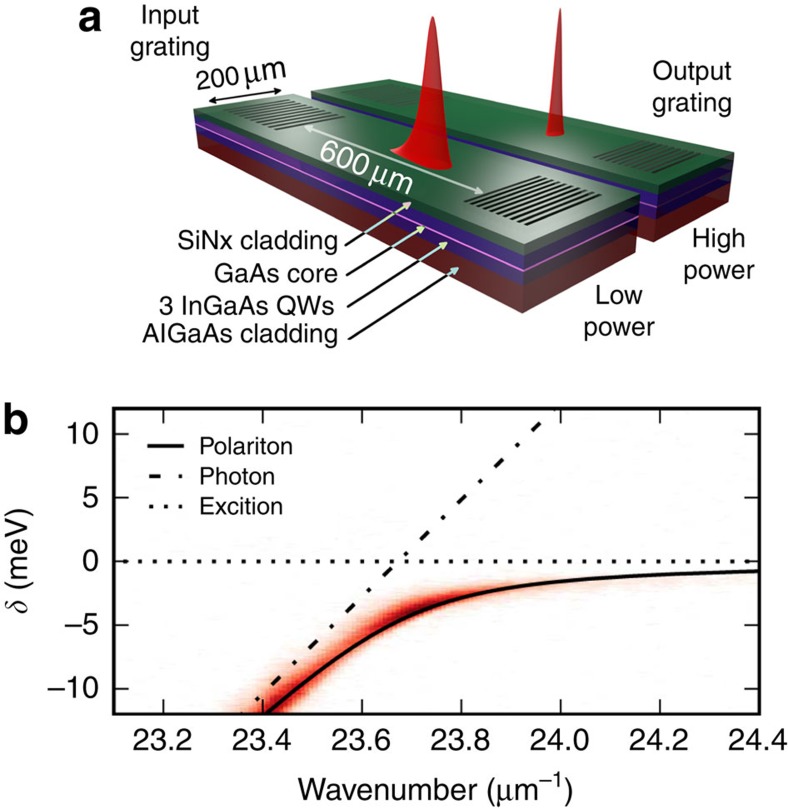
Exciton polaritons in waveguides. (**a**) Schematic diagram of waveguide (the same waveguide is shown with pulses under low-power and high-power excitation conditions). (**b**) Angle-resolved photoluminescence spectrum showing emission from the lower polariton branch in red. The fitted polariton dispersion (solid line) is shown with the uncoupled exciton and photon modes, which would exist for zero light–matter coupling (dotted and dashed lines). The horizontal and vertical axes are respectively the wavenumber of the guided mode and the detuning *δ* between polariton and exciton energies.

The structures we use in this work are planar AlGaAs waveguides with quantum wells embedded in the core parallel to the waveguide plane^25^ (see schematic diagram in Fig. 1a). Diffraction in the crystal growth direction is prevented by total internal reflection. As the photonic losses are weak and broadening of the exciton line is suppressed (the temperature is 10 K), the system is in the strong coupling regime and exciton-polaritons are formed. The sample is excited by pulses with a square spectral envelope of 5.5 meV corresponding to an ∼350 fs temporal full width at half maximum (FWHM). The pulses are coupled in through the input grating, propagate 600 μm and couple out through the output grating (see Methods, Supplementary Note 1 and Supplementary Fig. 1). Figure 1b) shows the polariton dispersion relation determined by angle-resolved photoluminescence spectroscopy^25^. As the momentum increases along the lower polariton branch, the polaritons become more matter-like and their group velocity approaches zero.

### Theoretical description

To model the experimental results, we describe a light pulse interaction with a narrow exciton resonance using the Maxwell–Lorentz system. Any GVD related to the waveguide geometry is negligible over the spectral range close to the exciton resonance and in comparison with the GVD induced by the photon–exciton coupling. The resulting set of equations accounting for the dominant nonlinearity originating in the two-body exciton–exciton interaction is


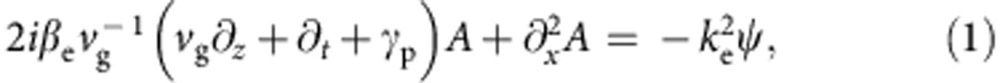



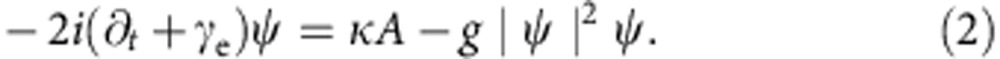


Here *z* and *x* are the coordinates, respectively, along and across the propagation direction and *t* is time. *A* is the amplitude of the photonic mode and *ψ* is the excitonic polarization scaled to be in the same units as *A*. Other symbols are defined in the methods. Writing *A* and *ψ* as plane waves allows us to obtain the polariton dispersion law, which relates frequency and wavenumber and is shown as a solid line in Fig. 1b (see Methods). The derivatives of this dispersion law fully characterize the dispersion experienced by the pulses as they propagate in the waveguide (see Supplementary Note 1). In our experiment, the pulse central (carrier) frequency ℏ*δ*_c_ varies from −7.6 to −10.4 meV and we obtain a positive GVD parameter *β*_2_ between 400 and 1,000 ps^2^ m^−1^, which corresponds to very strong normal dispersion (see Methods). The dispersion lengths are in the range ≃44–110 μm; hence, we observe the pulses after they have travelled between 5.5 and 13.6 dispersion lengths in the waveguide. The excitonic content of the polaritons is in the range 16% to 26%.

Within our experimental and theoretical frameworks, we study both quasi-one-dimensional effects, where diffraction along *x* is not important, and two-dimensional effects, where diffraction is relevant for the physics of the process. Mathematically, the system is similar to the equations used to study gap solitons^4^ and solitons with dispersion provided by material resonances^29^,^30^,^31^,^32^. Spatio-temporal solitons are a separate large area of research, where the most convincing experimental measurements of quasi-solitonic effects were made in *χ*^(2)^ crystals with very challenging dispersion control^33^ and in arrays of coupled waveguides^34^,^35^.

### Temporal solitons

**Figure 2:**
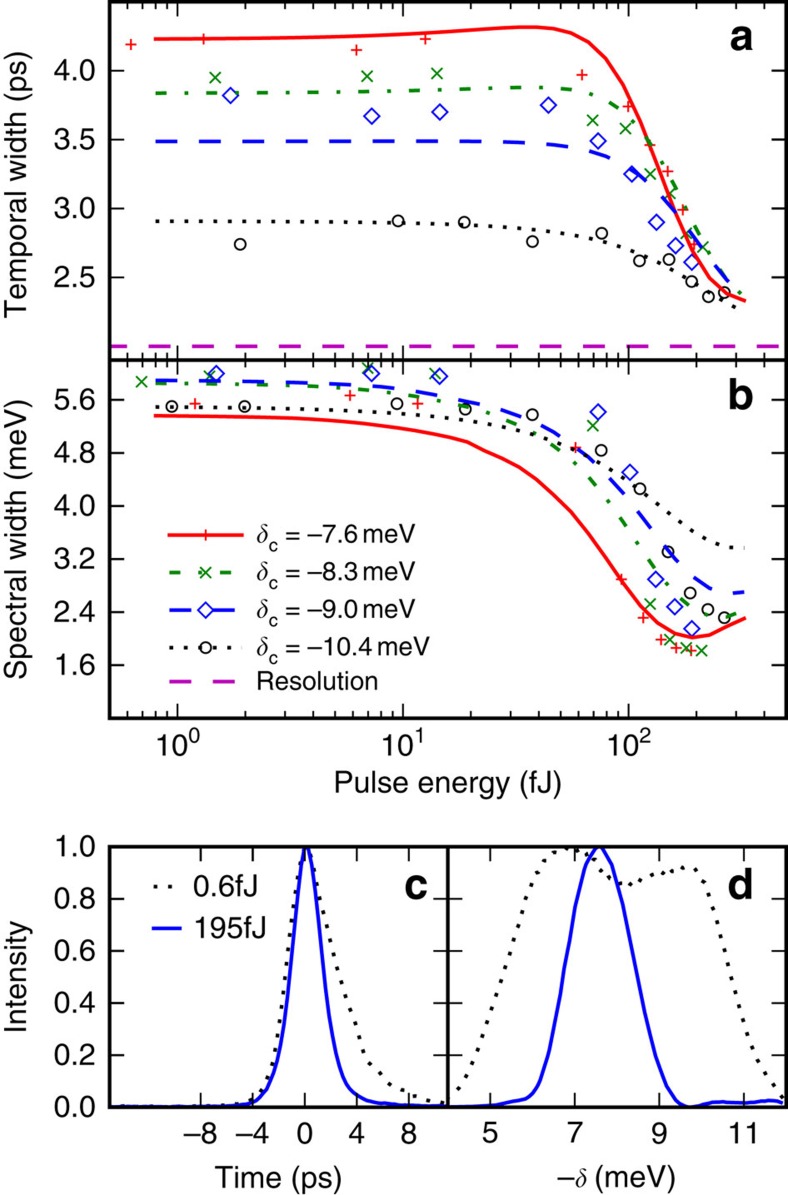
Comparison of experimentally measured and numerically simulated temporal and spectral pulse FWHMs at the output grating. (**a**) Experimental (symbols) and numerically simulated (line) pulse temporal duration versus pulse energy inside the waveguide for a number of detunings *δ*_c_ of the central pulse frequency from the exciton. (**b**) Experimental (symbols) and numerically simulated (lines) pulse spectral width versus pulse energy for the same detunings. (**c**) Temporal profiles at high and low pulse energy. (**d**) Spectral profiles at high and low pulse energy. Uncertainties of 1% in the experimental points are due to reading the FWHM from the noisy curves. Numerical simulations are for similar excitation conditions as in the experiment. The semi-quantitative agreement indicates that solitons are observed in the experiment.

We proceed by describing our results on observation of one-dimensional temporal solitons. We first present experimental measurements, which demonstrate temporal soliton formation and then confirm this by comparing the data with numerical modelling. Dependencies of the output pulse width versus total output pulse energy are shown in Fig. 2a) for several detunings of the central pulse frequency from the exciton, *δ*_c_. The detector resolution causes the incident 350 fs pulse to be measured as 2.0 ps. Below a threshold pulse energy, the measured FWHM after propagation through the waveguide is between 2.7 and 4.2 ps, depending on *δ*_c_, which indicates significant temporal speading due to the GVD. Figure 2c shows the temporal profile of the pulse collected from the output grating for pulse energies above and below the threshold. One should note that the pulse shape is highly asymmetric below threshold and becomes symmetric above threshold. Figure 2b shows the corresponding output spectral widths, whereas Fig. 2d shows pulse spectra above and below threshold. For low-pulse energies, the output spectrum reflects that of the incident pulse slightly modified by the frequency-dependent coupling efficiency of the grating coupler (see Supplementary Note 2 and Supplementary Fig. 2). As the pulse energy is increased above threshold, the pulse becomes significantly shorter and more symmetrical in time until, for the strongest pulses, the measured FWHM reaches a minimum between 2.4 and 2.7 ps, depending on *δ*_c_, as seen in Fig. 2a. At the same time, the spectrum shows strong narrowing about the central frequency reaching values in the range 1.8 to 2.3 meV, as shown in Fig. 2b. During this process, the total power collected from the output grating remains linear with pump power to within a few per cent, indicating that the spectral and temporal compression are due to a coherent transfer of energy between frequency components rather than a nonlinear absorption or filtering effect. Such coherent spectral and temporal compressions occuring at a critical input energy and levelling off with further energy increase are characteristic of the transition from dispersive propagation to soliton formation. The spectral compression occurs because of the presence of a small amount of linear loss (see Supplementary Note 3 and Supplementary Fig. 3).

**Figure 3:**
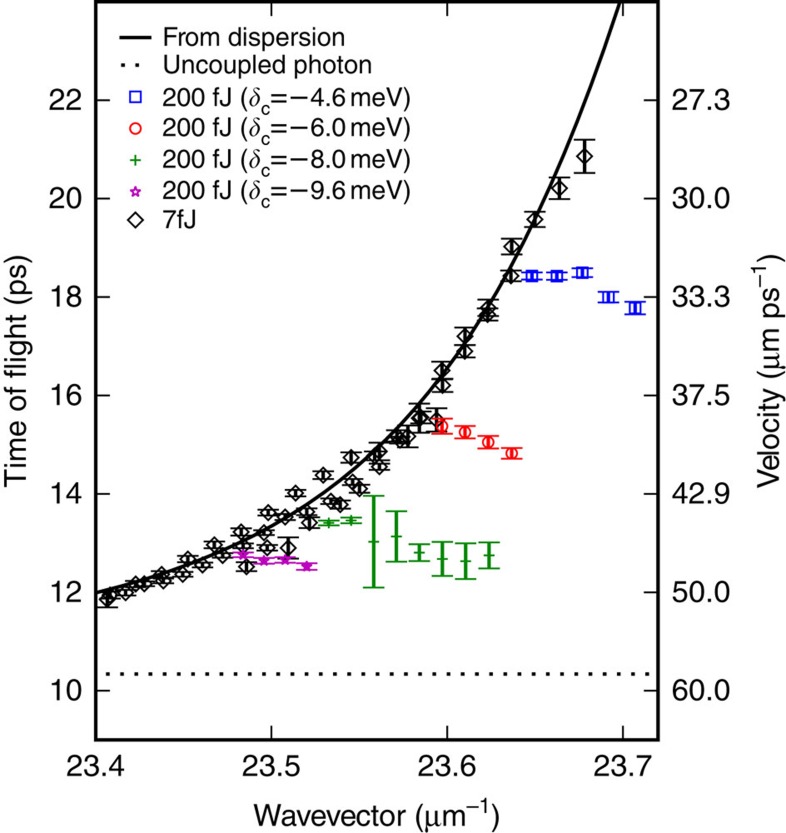
Measured time of flight of different wavevector components of pulse at low power (black diamonds) and high power (coloured symbols), and time of flight extracted from curvature of polariton dispersion (solid curve). The error bars give the 95% confidence interval for the time of flight and were calculated from the uncertainties in positions determined by fitting the incident and transmitted pulses (see Methods).

Another important evidence for solitonic propagation is that GVD is cancelled by self-phase modulation so that there is no temporal offset between spectral components of the soliton. We will now demonstrate that the above threshold pulses in our system are solitons by showing that all their spectral components travel at the same velocity. Figure 3 shows the time of flight of different spectral (wavevector) components of the pulse above and below threshold (see Supplementary Note 1). Below threshold, the time of flight is simply determined by the polariton dispersion relation with polaritons close to resonance travelling slower (see Fig. 1b). Above threshold, however, there is a very clear departure from this trend. The arrival time of the different spectral components is nearly independent of wavevector. This should be compared with a spread of several picoseconds over the same wavevector range below threshold. The dispersion has therefore been cancelled out above threshold, confirming the formation of a soliton. For the range of measured detunings, the soliton velocity of 33–50 μm ps^−1^ is faster than that of low-power polaritons at the same wavevectors but significantly slower than the 58 μm ps^−1^ expected for uncoupled photons. The soliton velocity also depends strongly on detuning from the exciton. These facts confirm that the system remains in the strong coupling regime during the propagation of the high power pulse (see Supplementary Note 4). To summarize, the experimental evidence for soliton formation consists of strong coherent temporal and spectral narrowing of the output pulses at a critical energy and near independence of velocity among different wavenumber components of the pulses. These effects are seen for a range of detunings. As discussed above, the dispersion length varies by a factor of 2.5 over the range of detunings used. Thus, we observe that above threshold the dispersion is cancelled at a range of positions relative to the dispersion length and not just at a single position.

### Size of nonlinearity

Soliton formation requires the characteristic length scales for the buildup of nonlinear and dispersive phases to be equal. By comparing standard expressions for the two (see Methods), we are able to deduce the nonlinear parameter^36^ *γ*=−18,000 (Wm)^−1^ and an effective nonlinear refractive index for our waveguide *n*_2_=−1.6 × 10^−14^ m^2^ W^−1^ (see Supplementary Note 5). A similar value for *n*_2_ was obtained from the blueshift of the polariton dispersion above threshold (see Supplementary Note 6 and Supplementary Fig. 4). It is three orders of magnitude larger than 1.82 × 10^−17^ m^2^ W^−1^ in planar AlGaAs waveguides in the weak coupling regime^37^ and 6 × 10^−18^ m^2^ W^−1^ in silicon^17^,^21^ and InGaP^19^, which have recently been used in a suspended membrane photonic crystal geometry in two of the most promising demonstrations of solitons for integrated optics until now (further comparison in Supplementary Note 7). Our value of the nonlinearity is also in good agreement with other GaAs-based systems in the strong coupling regime, namely with polariton interaction strengths in Bragg microcavities^38^ (see Supplementary Note 6).

### Comparison with theory

**Figure 4:**
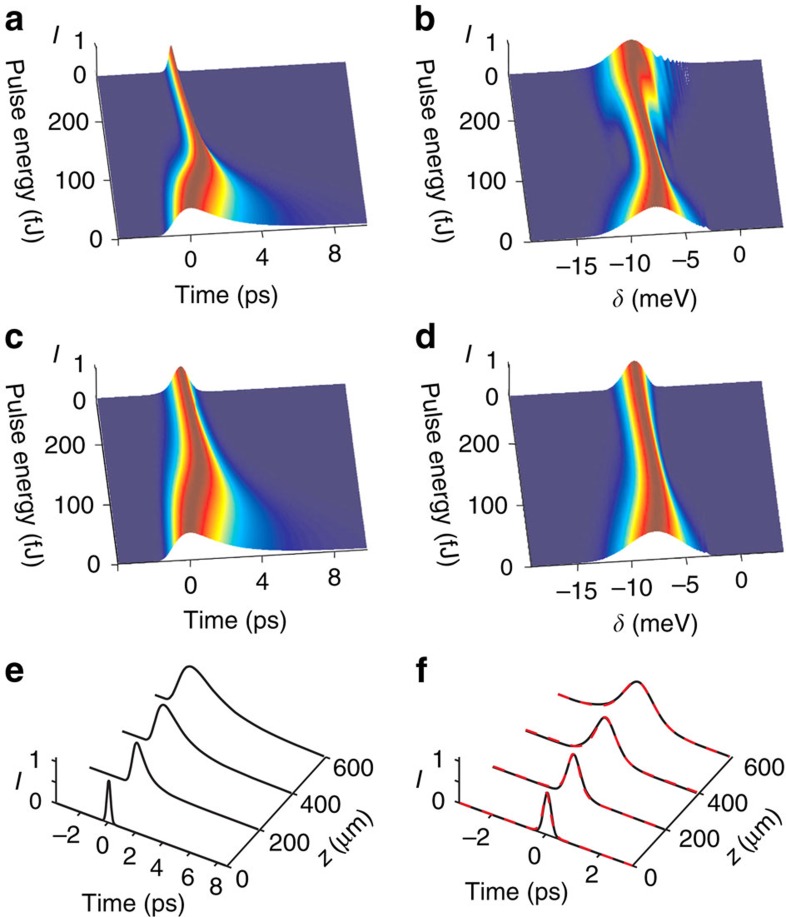
Numerically computed pulse propagation. Time and frequency domain pulse profiles at the end of the waveguide for the lossless (**a**,**b**) and lossy (**c**,**d**) cases. Evolution of the pulse along the length of the waveguide incuding loss for pulse energies below (**e**) and above (**f**) the soliton formation threshold, respectively. In **f**, the dashed red curves are analytic soliton solutions.

Figure 4a,b show numerically computed time domain and spectral pulse profiles versus pulse energy at the end of the waveguide under the assumption that loss is neglected. By tracing the evolution of the pulse along the waveguide, we have confirmed that exact temporal solitons are excited for pulse energies above ≃70 fJ. Experimental values of the losses introduced into the modelling modify the pulse dynamics only quantitatively (see Fig. 4c,d). They accelerate pulse broadening and shift the threshold for formation of solitons towards higher energies, ≃120 fJ. Figure 4e,f show evolution of the pulse profile along the waveguide with the real losses below (Fig. 4e) and above (Fig. 4f) the soliton formation threshold. For every profile shown in (Fig. 4e,f), the dashed red curves indicate a matching analytical soliton solution (see Methods), thus confirming that we are dealing with adiabatically decaying solitons. For comparison with experimental results, the modelled dependencies of the temporal and spectral widths of the output pulses on pulse energy are shown in Fig. 2a,b. The 2-ps resolution of the streak camera has been included in the temporal widths. The good semi-quantitative agreement between experiment and theoretical soliton solutions further confirms the observation of temporal solitons.

### Spatio-temporal solitons

**Figure 5:**
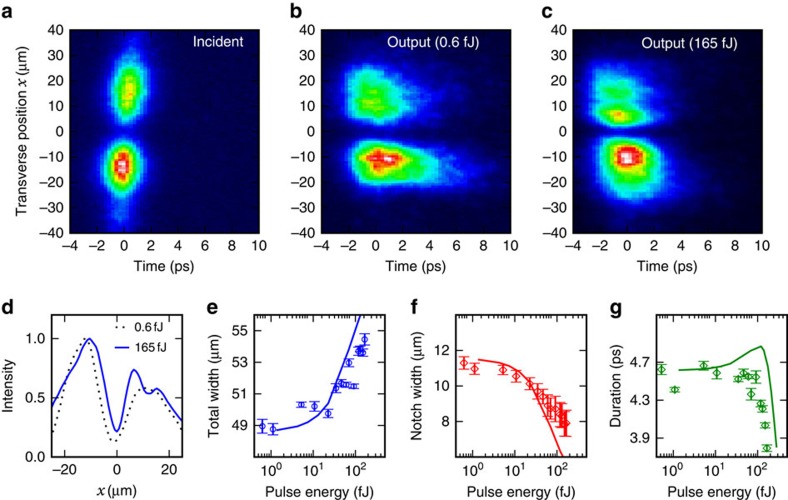
Evolution of the spatio-temporal wavepacket. Intensity profiles of the incident wavepacket (**a**) and the wavepacket after propagating through the waveguide at low (**b**) and high (**c**) powers as a function of time and transverse spatial direction *x*. (**d**) Cross-sections along *x* corresponding to **b** and **c**. Experimental (points) and simulated (lines) spatial widths of the total transverse distribution (**e**), dark notch (**f**) and temporal FWHM (**g**) as a function of pulse energy. The error bars give the uncertainty in estimating the widths from the positions of the half-maxima points and were calculated from fits of the experimental pulse spatial profiles.

We now shift our attention to the conditions where the wavepacket is strongly modulated in the transverse direction and show that the system supports dark–bright spatio-temporal solitons. These are two-dimensional wavepackets, which simultaneously show dark soliton behaviour in the transverse spatial dimension^39^,^40^ and bright soliton behaviour in the temporal dimension. Figure 5a shows the intensity distribution of the wavepacket incident on the input grating recorded by imaging the input grating onto the entrance slit of the streak camera (see Supplementary Note 1). The distribution along *x* has two lobes separated by a dark notch in intensity at *x*=0 where the field undergoes a *π* phase jump, (see Supplementary Note 8 and Supplementary Fig. 5), which is the necessary initial condition for the injection of a single dark soliton^4^,^39^,^41^.

The wavepacket after propagation is shown for low and high power in Fig. 5b,c, respectively. As in the temporal soliton case discussed above, at low power the bright polariton field has spread in time due to dispersion, whereas at high power the spreading is cancelled. Figure 5d shows cross-sections along *x* of the wavepackets in (Fig. 5b,c). The widths of the distribution and of the dark notch are summarized in Fig. 5e,f, respectively. As the pulse energy increases, it can be seen that the notch narrows by 30%, while the total width of the distribution increases by 10%. The width of a dark soliton is determined by the background intensity, which is analagous to quantized vortex cores in optical superfluids whose sizes are determined only by the fluid density^42^,^43^. In our system, the narrowing of the dark notch therefore represents the distribution moving towards that of the ideal dark soliton as the pulse energy is increased^41^. At the same energies at which the spatial soliton forms, the temporal duration of the pulses summarized in Fig. 5g decreases from 4.7 to 3.6 ps. Thus, we have observed clear experimental signatures of spatio-temporal dark–bright solitons. These solitons have been previously studied only theoretically^37^,^44^. We have also numerically modelled the evolution of the spatio-temporal wavepackets and found our results to be in good agreement with the experiment. The numerically computed spatial and temporal widths are shown as solid lines in Fig. 5e–g. As in the experimental case, the nonlinear interaction widens the total distribution and narrows the dark notch by several microns with the rate of change of width increasing with power. The semi-quantitative agreement of these trends again confirms that the experiment observes spatio-temporal solitons. We note that our observation of two-dimensional effects is possible, because our system allows short nonlinear and dispersion lengths to be achieved without confining the optical mode in the *x* direction.

## Discussion

In conclusion, we have demonstrated formation of picosecond light–matter solitons with pulse energy of <0.5 pJ. We have studied both temporal and spatio-temporal nonlinear effects and observed dark–bright spatio-temporal solitons. The solitons exist in a novel regime where intrinsically large dispersion is balanced by a nonlinearity several orders of magnitude larger than in any other ultrafast system. These properties allow nonlinear phases to accumulate and be controlled over short distances consistent with on-chip applications at low power. The waveguide exciton-polariton system therefore represents an important platform for high-speed integrated optics requiring large nonlinearity. We note that although the GaAs-based system we report requires cooling to 10 K, future polariton-soliton waveguide devices may exploit wide bandgap materials such as GaN (ref. 45) and ZnO (ref. 46) in which strong coupling and nonlinearities are robust at room temperature. It may be significant for these prospects that long-range polariton propagation in the linear regime has already been demonstrated in GaN quantum well waveguides^47^.

## Methods

### Experiment

The waveguide sample used in this work is the same as in ref. 25, except for the use of three quantum wells. The transverse-electric-polarized, guided optical mode couples strongly to the quantum well 1 s heavy-hole exciton to form transverse-electric-polarized polaritons with a vacuum Rabi splitting of 9 meV. The nearly frequency independent linear loss length of 400 μm corresponds to a polariton lifetime of ∼10 ps, which depends on detuning through the polariton group velocity. Losses are due to a combination of photon tunnelling through the cladding and optical absorption by the GaAs core material and tail of the exciton line. The loss length was measured by generating polaritons with an above-bangap laser and measuring the falloff of power emitted from an adjacent outcoupling grating with increasing source-detector distance (see ref. 25 for more details). In this work, excitation laser pulses were generated by spectrally filtering 100 fs pulses from a mode-locked Ti:Sapphire laser (see Supplementary Note 2) and injected into the waveguide by focussing the laser beam through a microscope objective onto the grating couplers (see Supplementary Note 1). The correct wavevector and energy for excitation were selected by tuning the incidence angle and central energy to correspond to those of the guided mode (see Supplementary Note 1). Coupling was maximized at around 12° by monitoring the emission from the output grating. Light coming from the sample was collected by the microscope objective and spatially filtered so that only light from the output grating was detected. The temporal profile of the pulses was measured by projecting an image of the output grating onto the entrance slit of a streak camera. The streak camera has a temporal resolution of 2.0 ps, measured using the reflection of the excitation laser pulse from the sample surface. The spectrally resolved emission was measured by projecting the fourier plane of the objective onto the entrance slit of an imaging spectrometer and integrating the light with a CCD (charge-coupled device) detector. The pulse energy was determined by focussing all light from the output grating onto a commercial photodiode-based power meter and dividing the average beam power by the laser repetition rate of 80 MHz. A ratio of 1:4 for the power scattered towards the power meter and that lost in the substrate was determined by modelling pulse outcoupling through the grating using a two-dimensional finite-difference-time-domain method and has been included to obtain the total pulse energy before outcoupling. The wavevector-resolved time-of-flight measurements were performed by projecting the fourier plane of the objective onto the entrance slit of the streak camera. The final lens was scanned, to align each wavevector component with the slit (see Supplementary Note 1).

### Theoretical model and polariton dispersion

In equations (1) and (2), *β*_e_ and *v*_g_ are the propagation constant and the group velocity of the photonic waveguide mode at the exciton frequency *ω*_e_=2*πc*/839.6 nm, *κ* is the rate of the light–matter coupling and *k*_e_=*ω*_e_/*c*. These were obtained from the fit to the measured polariton dispersion relation in Fig. 1. The loss rates *ℏγ*_p_=44.3 μeV and *ℏγ*_e_=7.5 μeV were chosen to fit the experimentally measured frequency dependence of the loss length. Taking plane waves (*A*, *ψ*)∝*e*^*iQz*−*iδt*^ and neglecting nonlinearity the lossless polariton dispersion relation from equations (1) and (2) is 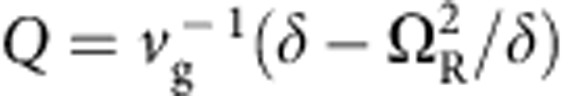. Here *δ* is a frequency offset from *ω*_e_, *Q* is a wavevector offset from *β*_e_ and 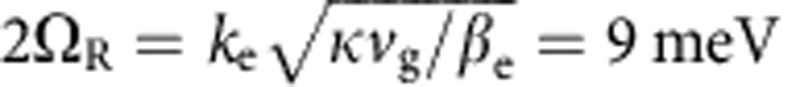 is the vacuum Rabi splitting. The derivatives of this dispersion law give the dispersion parameters for pulse propagation in the system. In particular, the GVD parameter 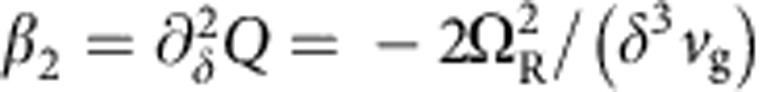. This has the opposite sign to *δ* so that the dispersion is normal (*β*_2_>0) for frequencies corresponding to the lower polariton branch (*δ*<0) and anomalous (*β*_2_>0) otherwise. The dispersion length is given by 
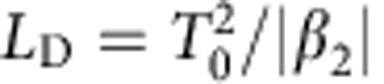
 where *T*_0_≃1.665/Δ*ω* is obtained from the pulse spectral FWHM Δ*ω*. The nonlinear length is given by *L*_NL_=1/(*γP*_0_)≈*T*/(*γE*) where *P*_0_ is the pulse peak power, *T*=350 fs is the pulse FWHM, *E* is the pulse energy and *γ* is the nonlinear waveguide parameter^4^,^36^. The nonlinear parameter may be related to the excitonic nonlinearity *g* in equations (1) and (2) by 
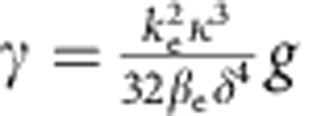
 when the the squared fields are in units of power.

### Analytical soliton solution

One-dimensional temporal solitons can be found analytically. Neglecting losses, *γ*_p_=*γ*_e_=0, and disregarding diffraction 
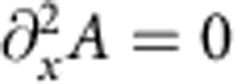
, the equations (1) and (2) become


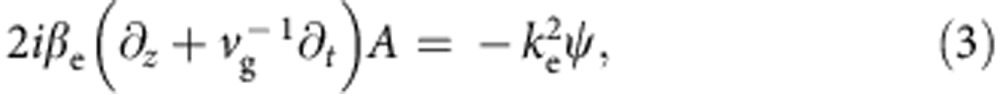



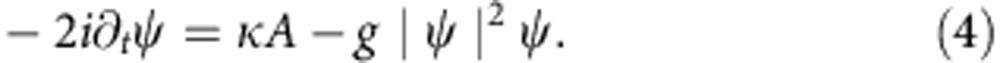


The conservative soliton solutions are parameterized by the soliton frequencies *δ*_s_ and velocities *v*_s_, and can be sought in the form


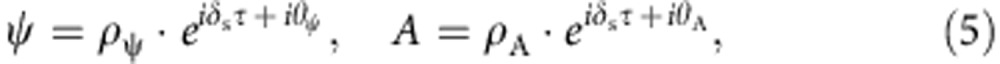


where *ρ*_ψ_(*τ*), *ρ*_A_(*τ*), *θ*_ψ_(*τ*) and *θ*_A_(*τ*) are real functions of *τ*=*t*−*z*/*v*_s_. Substituting the anzats (equation (5)) into equations (3) and (4), we obtain the expressions for amplitudes *ρ*_ψ_, *ρ*_A_ of the field. They read


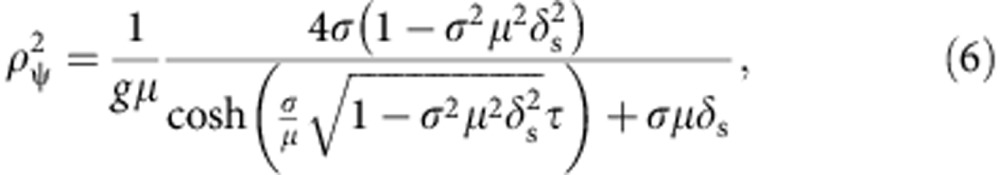



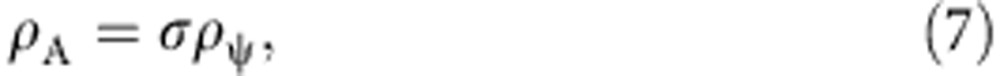


where 
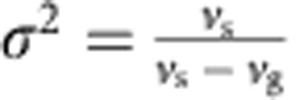
 and 
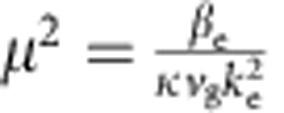
.

The expressions for the phases *θ*_ψ_ and *θ*_A_ of the fields are given by the integrals


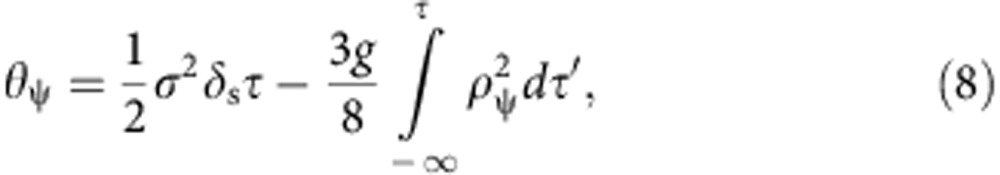



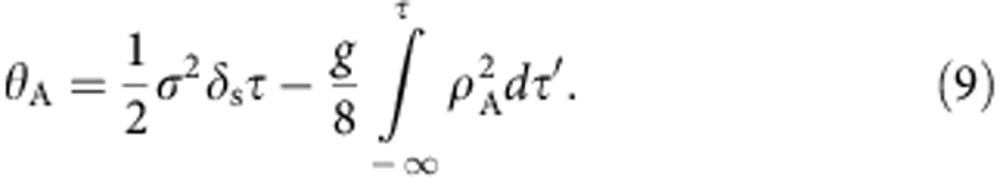


## Additional information

**How to cite this article:** Walker, P. M. *et al.* Ultra-low-power hybrid light–matter solitons. *Nat. Commun.* 6:8317 doi: 10.1038/ncomms9317 (2015).

## Supplementary Material

Supplementary Information Supplementary Figures 1-5, Supplementary Notes 1-8 and Supplementary References.
