# Supplementary material for: Ultra-low-power hybrid light–matter solitons
Source: Nat Commun. 2015 Sep 24;6:8317. doi: 10.1038/ncomms9317 (PMC4667441; doi:10.1038/ncomms9317)
Supplement: Supplementary Information — Supplementary Figures 1-5, Supplementary Notes 1-8 and Supplementary References. [file ncomms9317-s1.pdf]

Supplementary Figures

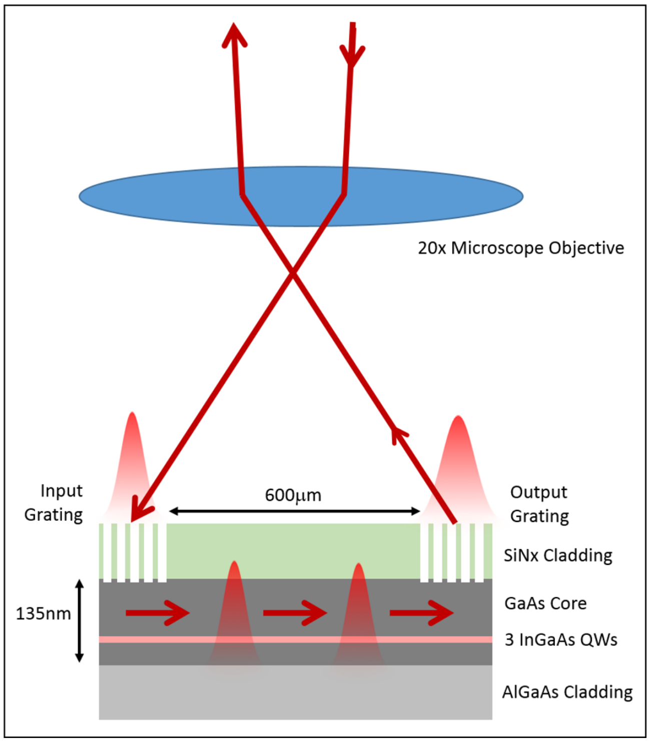

Supplementary Figure 1: Schematic diagram of light injection and collection. Light is focussed by the objective into the input grating, propagates through the waveguide core, exits through the output grating and is re-collimated by the objective.

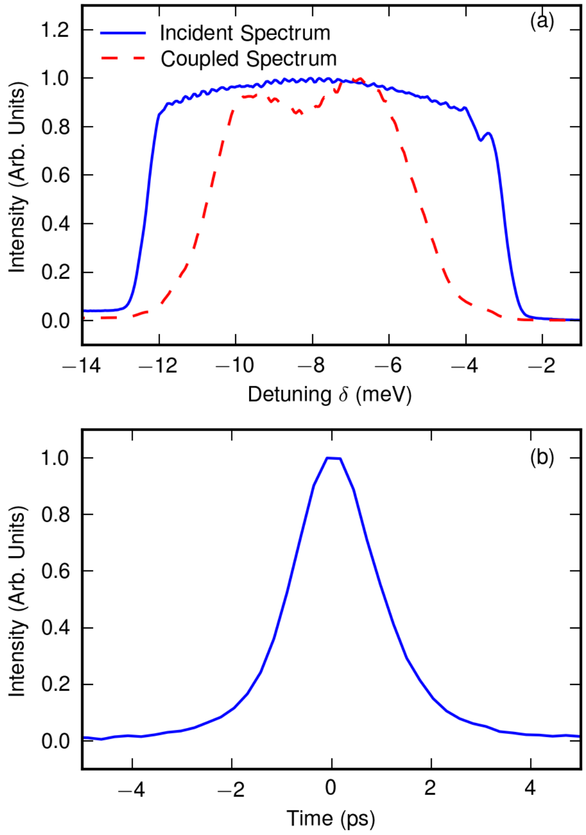

Supplementary Figure 2: Properties of incident and coupled pulses. (a) Incident pulse spectrum and pulse spectrum after filtering by grating incoupler. (b) Streak camera measurement of incident pulse.

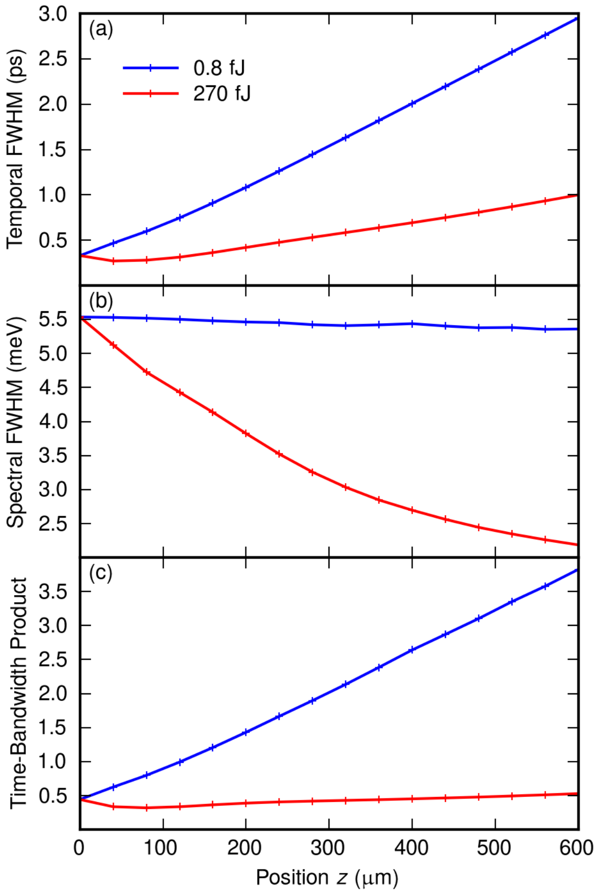

Supplementary Figure 3: Calculated evolution of pulse temporal (a) and spectral (b) width and the time-bandwidth product (c) with propagation along the waveguide in the linear (0.8fJ pulse) and soliton (270fJ pulse) regimes.

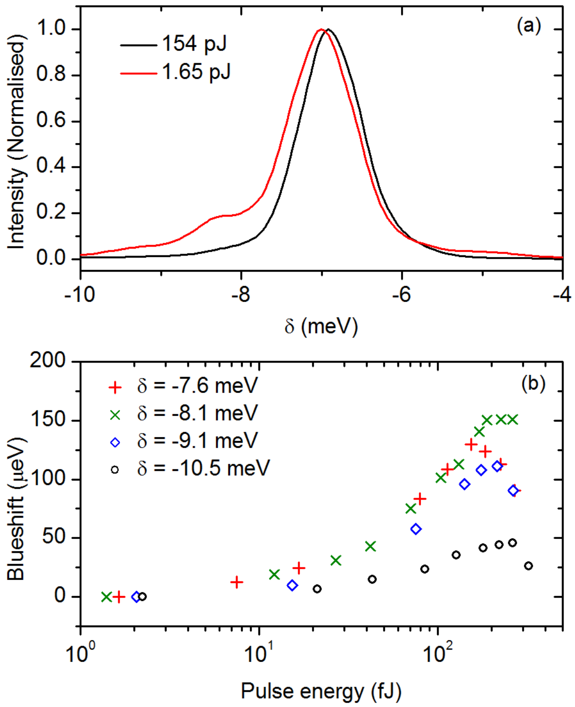

Supplementary Figure 4: Nonlinear interaction induced blueshift of polariton dispersion. (a) shows the spectra above and below threshold at the wavevector corresponding to the centre of the spectrum. (b) Frequency shift at the central wavevector as a function of total pulse energy.

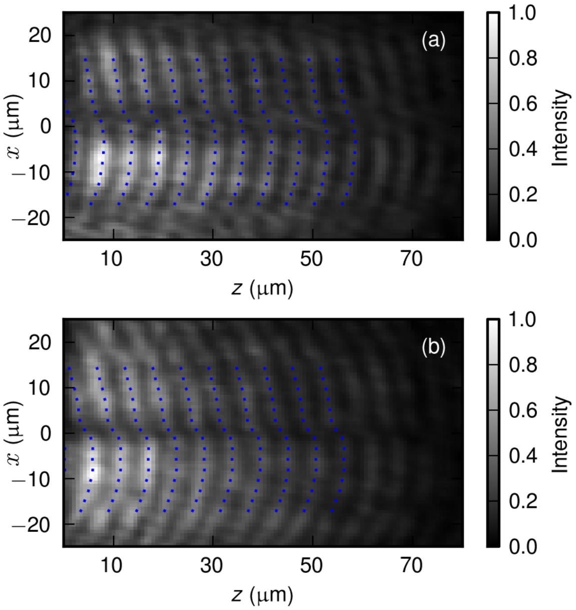

Supplementary Figure 5: Spatial phase profile at output grating at low (a) and high (b) excitation power. Blue dots are a guide to the eye.

Supplementary Notes

Supplementary Note 1 Grating Coupling and Dispersion Relation

Supplementary Figure 1 shows a schematic of the light injection and collection scheme. Incident light is focussed by a microscope objective onto a grating coupler. The grating couplers consist of a periodic modulation of the surface depth with a period of 0.25 μm over a distance of 200 μm in the propagation direction z. They are homogeneous in the direction transverse to propagation and 200 μm wide. The light is incident at an angle $\theta \sim$ -11.7 degrees from the sample surface. The angle may be freely adjusted by translating the beam before the objective perpendicular to the optic axis. The grating coupler acts like a diffraction grating and adds multiples of the grating wavevector $G = 2\pi/0.25$ μm = 25.1 μm^{-1} to the free-space wavevector $k_z = (2\pi/\lambda)\sin(\theta)$. Only the zero order diffraction is supported in air since the total possible wavevector in air at the operating wavelength around 841.5nm is 7.47 μm^{-1}. The +1 diffraction order couples the light to the waveguide guided mode which has a propagation constant of 23.6 μm^{-1} at the operating wavelength. There is no guided mode at the wavevector of -26.6 μm^{-1} corresponding to the -1 diffraction order. Thus the incident light may be either reflected, transmitted or couple to the guided mode in the forward direction. The exact angle of incidence is chosen to maximise light coupling to the guided mode at the wavelength currently in use. The incident beam had no wavevector component perpendicular to the propagation direction z between the centres of the input and output grating couplers so that the light travelled directly between the centers of the gratings.

In the region of the output grating the decay length of the polaritons is much shorter than in the unpatterned waveguide because they may tunnel out into

free space photon modes above and below the waveguide. Outcoupled light is collected by the same microscope objective used to excite the system. Direct imaging of the output grating onto a CCD camera reveals that the decay length of polaritons on the grating is $\simeq$ 20 μm. This is ten times shorter than the length of the grating so that we can be sure all the light is coupled out from the guided mode. Imaging the grating onto a CCD camera also allows the spatial intensity profile transverse to the propagation direction to be measured directly. Examples of the spatial profile measured in this way are shown in Fig. 5 (a-d) in the main text where the narrowing of the spatial dark notch indicates dark soliton formation. For the data in Fig. 5 the temporal profile is recorded simultaneously with the spatial profile as the CCD camera is attached to the exit of a streak camera. The sample was aligned so that the direction transverse to propagation, x, was parallel to the streak camera entrance slit.

As with the input grating, the output grating diffracts the guided mode out to free space with each wavevector (propagation constant) inside the waveguide corresponding to a different angle in air. By imaging the Fourier plane of the objective onto a detector we are able to record wavevector resolved images of the light in the waveguide. In Fig. 1(b) in the main text this is used to make wavevector and energy resolved photoluminescence images which reveal the polaritonic dispersion relation of the guided mode. In Fig.3 in the manuscript this technique is used to produce wavevector resolved time-of-flight measurements. The time of flight was determined at a series of wavevectors by scanning the image of the fourier plane across the streak camera entrance slit. For each wavevector the reflection of the incident laser pulse from the input grating and the pulse coupled out from the output grating after propagating through the waveguide were simultaneously recorded on the streak camera. The peaks were fit with gaussians and the time of flight between them extracted.

Since the guided mode dispersion provides a one-to-one mapping of wavevector to frequency this technique allows us to measure the temporal spread among different frequency components of a pulse, which is a measure of the propagation induced chirp.

The measured polariton dispersion relation (Fig.1(b) in the main text) provides the full relationship between frequency and propagation constant (wavevector) for light in the waveguide. It allows complete characterisation of the size of the GVD without the need to compare input and output pulse widths. The expression for the dispersion relation is given in the methods in the main text and it is plotted as the solid black curve in Fig. 1(b) where the good agreement with the measured PL spectrum can be seen. The dispersion parameters β_2 etc., usually used to characterise dispersive pulse spreading [1], are simply the values of the derivatives of this relation taken at the central pulse frequency. The fact that these correctly describe the GVD experienced by the pulses in the waveguide is shown by the wavevector resolved time of flight measurements taken at low power, shown in Fig.3 in the main text. The solid line shows the expected spread of arrival times calculated from the dispersion relation and agrees well with the measured spread in arrival times (black points). The expression for the second order dispersion coefficient β_2 in terms of detuning of the pulse central frequency from the excitonic resonance is given in the methods in the main text. Using the expression we obtain β_2 between 400 and 1000 $\mathrm{ps}^2\mathrm{m}^{-1}$ for the detuning range we use as stated in the manuscript. The dispersion length, which characterises the spreading of a pulse with a particular spectral width, is given by $L_\mathrm{D} = T_0^2/\beta_2$ [1]. Here it is important to note that $T_0 = 1.665/\Delta\omega$ is properly obtained from the spectral FWHM $\Delta\omega$ of the pulse and not from the actual temporal profile. This is because dispersion is fundamentally the accumulation of phase differences between spectral components of a pulse. A

discussion of the propagation of pulses under the action of dispersion is available in Supplementary Reference [1]. Using our measured pulse spectral width of 5.5meV and the values of β_2 above gives $T_0 = 0.21$ps and we obtain dispersion lengths between 44 μm and 110 μm. Thus the measured spectral width and the measured polariton dispersion relation, confirmed by the low power time of flight measurements, fully quantify the dispersion.

Supplementary Note 2 Input Pulse Characteristics

Near transform-limited 100 femto-second pulses were obtained from a Spectra-Physics Tsunami mode-locked Titanium doped Sapphire laser. They were passed through a dispersion-free diffractive pulse shaper [2] with a mechanical variable slit to allow transform limited pulses of variable temporal width to be generated. The spectral envelope of the pulses matches the square transmission function of the slit.

Supplementary Figure 2(a) shows the pulse spectrum incident on the sample and the spectrum coupled in through the grating for a detuning δ_{c} = -7.6meV. The two are not the same because only those components of the pulse which match the angle and frequency of the guided mode within the waveguide will efficiently couple to it. Therefore the waveguide filters the incident spectrum and the coupled spectrum is narrower than the incident spectrum. The coupled spectrum was measured by collecting the spectrum from the output grating which is reasonable given that the loss length does not vary significantly with frequency over the pulse spectral range and the output coupler couples out all the light in the waveguide regardless of frequency (see Supplementary Note 1). Supplementary Figure 2(b) shows the temporal profile of the incident pulse corresponding to the spectra in Supplementary Figure 2(a) as recorded by the streak camera. The measured FWHM of the temporal profile of the input

pulse is $\Delta T_{\text{meas}} = 2$ ps. The real pulse temporal width may be obtained using $\Delta T = \sqrt{\Delta T_{\text{meas}}^2 - \Delta T_{\text{res}}^2}$ where ΔT_{res} is the streak camera resolution. In fact we measure $\Delta T_{\text{res}} = 2$ps so the incident pulse is indistinguishable from the streak camera response function. This shows that the input pulse is much shorter than 2ps and so it is reasonable to take the pulse temporal width of 350fs from the measured spectral width of the pulse.

Supplementary Note 3 Spectral Compression and The Effect of Loss

In Fig. 2 in the main text it is seen that for high energy pulses both the temporal and spectral widths narrow. In this section we will discuss the mechanism which selects spectral narrowing rather than spectral broadening or, indeed, spectral invariance. First we note however that although a simultaneous spectral and temporal narrowing might appear at first glance to violate the Fourier limit this is not the case. At low power the pulse observed at the output has undergone a large amount of dispersion and so its temporal width is expected to be much larger than the Fourier limit. Above threshold the dispersion is cancelled so the time-bandwidth product is much less than in the highly dispersive low power case.

We now discuss how the presence of weak linear losses leads to spectral compression. In general pulses may undergo either spectral compression or expansion or remain the same. In the most simple system, described by the Nonlinear Schrodinger Equation (NSE) without perturbations, an incident pulse which exactly matches the shape and magnitude of the fundamental soliton solution of the waveguide will propagate forever without changing its spectral or temporal distribution away from those of the incident pulse. Weak losses add an extra perturbative term to the NSE and the effect of this term on the solutions has

been much studied in the literature (See e.g. Supplementary References [1, 10]). Qualitatively, as the pulse propagates and loses energy the peak power and hence the rate of nonlinear phase shift decreases. To keep the nonlinear and dispersion lengths balanced the spectrum must narrow in order to reduce the rate of dispersive phase shift. The pulses remain close to the ideal (lossless) soliton solutions but undergo an adiabatic temporal spreading and spectral narrowing as they propagate. This process of adiabatic pulse adjustment to compensate the loss occurs while the loss length is larger than both the nonlinear and dispersion lengths, as in our case where $L_{\text{loss}} = 400\mu\text{m}$ (methods A) is longer than $L_{\text{NL}} = L_{\text{D}} \sim$44-100 μm.

We have confirmed that this behaviour is reproduced in our system using the numerical model. In Fig. 4(f) in the main text the gradual temporal spreading with propagation distance along the waveguide may be seen. In Supplementary Figure 3 we summarise the spectral and temporal widths and the time-bandwidth product of the pulse as it propagates in the linear and solitonic regimes. In the low power regime the spectral width is essentially unchanged apart from a small decrease due to a small frequency dependence of the loss length. The temporal width (not including convolution with the detector response) increases rapidly due to dispersion. In the high power case it can be seen that, as expected, the much less rapid temporal spreading is accompanied by a gradual spectral narrowing which keeps the time-bandwidth product as close to that of the ideal soliton solution as possible. The numerical results demonstrate that the coupled light-matter system behaves in a qualitatively similar way to the NSE with loss.

Supplementary Note 4 Strong Coupling in the Soliton Regime

Figure 1(b) in the main text shows the polariton dispersion relation measured at low power using continuous wave above-bandgap excitation. The strong coupling is shown by the curvature of the mode. For more details see Supplementary Reference [9]. For the pulsed excitation used in this work the strong coupling at low power is shown by the good agreement of the measured and modelled frequency dependent times of flight shown in Fig. 3 in the main text. It is known that at high intensities the excitons may be destroyed and strong coupling lost. It is important for the results presented in this paper that the strong coupling is maintained in the high power pulsed regime where solitons are observed. We show that this is the case in three ways.

Firstly, the observed energy blueshift of the polaritons at the output grating was 100 μeV in the soliton regime which is much less than half the vacuum Rabi splitting (4.5 meV). It corresponds to an exciton density of $3 \text{x} 10^9$ cm^{-2} in each quantum well (See Supplementary Note 6), which is more than an order of magnitude smaller than the Mott density at which the excitonic resonance collapses [4]. Secondly, we note that the dependence of output power on input power was linear. If the density were sufficiently high to bleach the exciton resonance one would expect the broadened exciton line to cause extra absorption leading to a sublinear output power dependence, which was not observed.

Finally, we examine the soliton velocities. In Fig. 3 in the main text we plot wavevector resolved time of flight measurements for a range of detunings. There is a very strong dependence of the soliton velocity on the detuning of the pulse central frequency from the exciton. This shows that the photons are still strongly coupled to the excitonic resonance even at high pulse powers. Additionally, as noted in the main text, the group velocities of the polaritons

are much less than the uncoupled photon group velocity of 58 μm ps^{-1} which one would expect if the system had reached the weak coupling regime.

In conclusion, for all the reasons discussed above e.g. small energy shift, lack of nonlinear absorption, strong detuning dependence of soliton velocity and soliton velocity lower than that of weakly coupled photons, we can be sure that the system remains in the strong coupling regime at high powers.

Supplementary Note 5 Deduction of Effective Nonlinear Refractive Index

Soliton formation requires the characteristic length scale $L_{\mathrm{NL}} = 1/(\gamma P_0) \approx T/(\gamma E)$ for the buildup of nonlinear phase to be comparable to the dispersion length $L_{\mathrm{D}} = T_0^2/|\beta_2|$. Here, P_0 is the pulse peak power, T=350fs is the pulse width, E is the characteristic pulse energy for the soliton formation, γ is the nonlinear waveguide parameter [1] and $T_0 \simeq 1.665/\Delta\omega$ is obtained from the pulse spectral FWHM $\Delta\omega$ (see Supplementary Note 1). We note that solitons in any lossy system will lose intensity as they propagate with a characteristic loss length L_{loss} which is 400 μm in our system. Provided that $L_{\mathrm{NL}} = L_{\mathrm{D}} < L_{\mathrm{loss}}$ the nonlinearity will adiabatically adjust the pulse width to compensate the loss and solitons may still be expected (see Supplementary Note 3). From these considerations we now estimate the nonlinear parameter and an effective nonlinear refractive index for our system. Taking the threshold pulse energy at the waveguide exit as 100fJ and L_{loss} = 400μm we estimate E = 450fJ at the input. Setting $L_{\mathrm{NL}} = L_{\mathrm{D}}$ we obtain $\gamma = T/(EL_{\mathrm{D}}) \sim$ -18,000/(Wm). This may be related to an effective nonlinear refractive index by the standard formula $\gamma = n_2 k_0/A_{\mathrm{eff}}$ where k_0 is the vacuum wavenumber and A_{eff} = 6.6 μm^2 is the effective mode area from Eqn. 1 [1].

$$A_{\text{eff}} = \frac{\left(\int\int_{-\infty}^{\infty} |F(x,y)|^2 \, dxdy\right)^2}{\int\int_{-\infty}^{\infty} |F(x,y)|^4 \, dxdy} \simeq \Delta x \Delta y \frac{\pi}{2ln(2)} \quad (1)$$

We obtain n_2 =-1.6x10^{-14} m^{2}W^{-1}. In using this expression for n_2 we have treated the waveguide with embedded quantum wells as an effective material. When comparing to waveguides composed of bulk materials the ratio of our n_2 to that for the bulk material gives the ratio of nonlinear phase shift for devices of the same physical size operating at the same wavelength and power density. In the above we have assumed that the pulse is transform limited which gives the maximum peak power P_0 for a given spectral width. For pulses longer than the transform limit the dispersion, given by the spectrum, would be the same but the peak power would be lower so that a nonlinearity larger than the value quoted would be needed to balance the dispersion.

Supplementary Note 6 Comparison of Nonlinearity with Bragg Microcavity Polaritons and Deduction of n_2 from Blueshift

The polariton nonlinearity in planar microcavities is expressed in the literature by the interaction induced frequency blueshift per exciton in one square micrometer of a single quantum well and is in the range 2-10 μeV μm^2 per exciton (see Supplementary Reference [3] and references therein). The very large nonlinearity in our system allows us to directly observe this blueshift in the emission from the output grating.

In Supplementary Figure 4 we plot the spectral shift of the emission at the central wavevector as the pulse energy is increased. There is a clear shift of the emission towards higher energies as the pump power increases. This blueshift increases linearly with pulse energy and once the energy is greater than the

soliton formation threshold it decreases. It is important to note that although the blueshift decreases the total output pulse energy remains linear with input power so that no polaritons are lost but are only transferred to different states. For pulse energies above threshold the blueshift decreases meaning that the peak density decreases. This is because for pulse energies above threshold the spectrum gradually becomes modulated, with particles being transferred into spectral sidebands. The detailed study of this effect is beyond the scope of this paper.

The pulse energy may be converted to a peak number density of excitons per quantum well using equation (2) where E is the pulse energy, Δx =20 μm is the transverse spatial FWHM of the soliton, $|X|^2 = 26\%$ is the excitonic content of the polaritons, v_{g} =43 μm ps^{-1} is the group velocity, T is the pulse temporal FWHM, ω is the pulse central frequency and $N_{\mathrm{QW}} = 3$ is the number of quantum wells and values are given for a central pulse frequency detuning $\hbar\delta_{\mathrm{c}}$ = -7.6 meV from the exciton.

$$N_{\mathrm{peak}} = |X|^2 \frac{E}{\hbar\omega} \frac{1}{v_{\mathrm{g}} T \Delta x N_{\mathrm{QW}}} \tag{2}$$

At the output grating the observed blueshift $\Delta E = 100$μeV for a pulse energy of 0.1 pJ. Taking the pulse width at the output as T=1.5ps we obtain N_{peak} = 28.5 excitons per μm^2 per quantum well which gives an interaction constant $g_{\mathrm{X}} = \Delta E/N_{\mathrm{peak}}$ = 3.5 μeV μm^2 which is in good agreement with a theoretical estimate $g_{\mathrm{X}} = 3a_{\mathrm{B}}^2 E_{\mathrm{x}}$ = 3 μeV μm^2 where a_{B} and E_{x} are the exciton bohr radius and binding energy respectively [3].

To compare the fundamental excitonic nonlinearity g_{X} common in exciton-polariton literature with the nonlinearity γ of nonlinear optics we note that a frequency blueshift at fixed wavevector and a wavevector shift at fixed frequency are related by the group velocity using equation (3)

$$\Delta k = \frac{dk}{d\omega}\Delta\omega = \frac{\Delta\omega}{v_{\mathrm{g}}} \tag{3}$$

The nonlinear length may then be obtained using $L_{\mathrm{NL}} = 1/\Delta k$. Combining these with equation (2) leads to the relation between g_{X} and γ given in equation (4).

$$\gamma = \frac{(g_{\mathrm{X}}/\hbar)\,|X|^2}{\hbar\omega\Delta x N_{\mathrm{QW}} {v_{\mathrm{g}}}^2} \tag{4}$$

Inserting the value $g_{\mathrm{X}} = 3.5$ μeV μm^2 obtained from the blueshift measurements we obtain $\gamma = 52800$ (Wm)$^{-1}$. To convert the nonlinear parameter γ to the nonlinear refractive index we use equation $n_2 = \gamma A_{\mathrm{eff}}/k_0$ where $A_{\mathrm{eff}} = 6.6$ μm^2 is given by equation (1) [1]. We obtain n$_2$ = 4.7x10^{-14} m^{2}W^{-1}.

This direct estimate of the nonlinearity is a factor of three larger than the value obtained in the main text using a comparison of dispersion and nonlinear lengths. The agreement is reasonable given the uncertainties in some of the parameters. Both estimates are sufficient to establish the very large nonlinearity of the system and to show the overall reliability of the deduction of n_2.

Supplementary Note 7 Comparison with Other On-Chip Soliton Schemes

In Supplementary Note 5 we deduce a value $\gamma \sim 18000/(\mathrm{Wm})$ for the nonlinear parameter and n_2 =-1.6x10^{-14} m^{2}W^{-1} for the effective nonlinear refractive index. This is more than three orders of magnitude larger than 6x10^{-18} m^{2}W^{-1} in silicon [5, 6] and InGaP [7] which have recently been used in a suspended membrane photonic crystal geometry in two of the most promising demonstrations of solitons for integrated optics until now. In those systems solitons are formed at pulse energies of 12pJ for InGaP [7] and 9pJ for silicon [5] which

are 1.3-1.4 orders of magnitude higher than 0.45pJ for the energy at the input grating in our system. We note that in those works the effective mode areas are smaller ($\sim$0.5µm^2) and the dispersion lengths are much longer (3.6mm and 1mm) than in our system both of which can be used to reduce the threshold, the latter at the expense of increased circuit size.

It is also important to compare our nonlinearity to III-V planar waveguides in the weak coupling regime. In Supplementary Reference [8] solitons were generated in a 2.5mm long AlGaAs waveguide at pulse energies of $\sim$40pJ and one may infer a nonlinear parameter $\gamma \sim 23/(\mathrm{Wm})$ which agrees with their quoted $n_2 =$ -1.82x10^{-17} m^{2}W^{-1} given the effective area 5 µm^2 and wavelength of 820nm. Thus, our nonlinearity is nearly three orders of magnitude larger than in very similar AlGaAs waveguides in the weak coupling regime.

Finally, it is worthwhile to consider how the soliton speeds in our system, which govern data transmission rate, compare with other systems suitable for on-chip soliton devices. The velocities in our system depend strongly on the detuning of the pulse from the exciton resonance and were in the range 32 to 49 µm ps^{-2}. These correspond to group indexes between 6.2 and 9.4 which are comparable to those in other systems suitable for on-chip soliton devices e.g. 8 and 30 for Supplementary References [7] and [5] respectively. One may also note that the group index is only a factor of $\sim$2 higher than 3.477 for bulk silicon at 1550nm which may be considered an upper limit on the speed of light for on-chip semiconductor photonic devices.

Supplementary Note 8 Spatial Soliton Phase

To produce the incident wavepacket for the spatio-temporal soliton experiments we used pulses with the same temporal profile as in the temporal soliton section. To introduce the spatial profile proper to a single dark soliton the excitation

laser beam was passed through a phase plate to produce a π phase jump at x=0. This follows the approach used in Supplementary Reference [11] for exciting one dimensional dark spatial solitons in a continuous wave system. The particular phase plate used in our case consisted of a thin glass microscopy cover-slip. One half was masked off using a photolithographic process and the other side etched in a reactive ion etcher to remove material corresponding to half a wavelength of light at 840nm. Since the light passing through one half of the plate travels further than light passing through the other half this results in a phase jump across a beam centered on the interface between etched and unetched regions.

In the spatio-temporal soliton experiments the laser pulses were passed through the phase mask and imaged onto the input grating. The presence of the π phase jump results in the dark notch in intensity appearing at the same position (x=0) as can be seen in Fig.5(a) in the main text. The phase profiles corresponding to the dark notch were also measured by interfering the light extracted from the output grating with a flat phase Gaussian reference beam. This was done using continuous wave (CW) excitation rather than pulsed because the very low average powers in the pulsed case required impractically long exposures compared to the interferometric stability of the system. The CW measurements are sufficient to characterise the spatial variation of the phase. The reference beam was taken from the same laser used to excite the sample at the input grating and expanded to form a large flat-phase reference spot on a CCD detector. Supplementary Figure 5 shows the interferograms for low power and high power excitation. In both cases the phase jump at x=0 is present at the output grating. In the high power excitation case the same narrowing of the dark notch and broadening of the bright background was observed as in the pulsed case presented in the main text. Thus the injected phase jump is present at the output for all powers beween the linear regime and powers where the dark

notch narrows.

Supplementary References

[1] Agrawal, G. P. Nonlinear Fibre Optics (Academic Press, 2001)

[2] Weiner, A. M., Heritage, J. P. and Kirschner, E. M. High-resolution femtosecond pulse shaping *J. Opt. Soc. Am. B* **5,** 1563-1572 (1988)

[3] Ferrier, L. et. al., Interactions in Confined Polariton Condensates *Phys. Rev. Lett.* **106,** 126401 (2011)

[4] Schmitt-Rink, S., Chemla, D. S. and Miller, D. A. B., Theory of transient excitonic optical nonlinearities in semiconductor quantum-well structures *Phys. Rev. B* **32,** 6601 (1985)

[5] Blanco-Redondo, A., Husko, C., Eades, D., Zhang, Y., Li, J., Krauss, T.F., and Eggleton, B.J, Observation of soliton compression in silicon photonic crystals *Nature Comms.* **5,** 3160 (Jan 2014)

[6] Zhang, J., Lin, Q., Piredda, G., Boyd, R. W., Agrawal, G. P. and Fauchet P. M. Optical solitons in a silicon waveguide *Optics Express* **15,** 7682 (2007)

[7] Colman, P., Husko, C., Combriê, S., Sagnes, I., Wong, C. W. and De Rossi, A., Temporal solitons and pulse compression in photonic crystal waveguides *Nature Photon.* **4,** 862-868 (2010)

[8] Bêlanger, N., Villeneuve, A. and Aitchison, J. S., Solitonlike pulses in self-defocusing AlGaAs waveguides *J. Opt. Soc. Am. B* **14,** 3003-3012 (1997).

[9] Walker, P. M. et. al. Exciton Polaritons in Semiconductor Waveguides *Appl. Phys. Lett.* **102,** 012109 (2012)

[10] Kivshar, Y. S. and Agrawal, G. P., Optical Solitons (Academic Press, 2003)

[11] Shandarov, V. Kip, D., Wesner, M. and Hukriede, J., Observation of dark spatial photovoltaic solitons in planar waveguides in lithium niobate *J. Opt. A: Pure Appl. Opt.* **2,** 500-503 (2000)
